# Nail and enthesis assessment in patients with psoriatic disease by high frequency ultrasonography: findings from a single-centre cross-sectional study

**DOI:** 10.1007/s11547-022-01568-4

**Published:** 2022-10-19

**Authors:** Piero Ruscitti, Maria Esposito, Camilla Gianneramo, Ilenia Di Cola, Andrea De Berardinis, Andrea Martinese, Gerard Nkamtse Tochap, Alessandro Conforti, Carlo Masciocchi, Paola Cipriani, Antonio Barile, Maria Concetta Fargnoli

**Affiliations:** 1 Department of Biotechnological and Applied Clinical Sciences, University of L’Aquila, Delta 6 Building, Via dell’Ospedale, PO box 67100, L’Aquila, Italy; 2 Dermatology Unit, San Salvatore Hospital, L’Aquila, Italy; 3 Department of Emergency and Interventional Radiology, San Salvatore Hospital, L’Aquila, Italy

**Keywords:** Psoriasis, Psoriatic arthritis, Musculoskeletal ultrasonography, High frequency ultrasound

## Abstract

**Purpose:**

To characterize nail and enthesis abnormalities using high frequency ultrasound (HFUS) in patients with psoriasis (PSO), psoriatic arthritis (PSA) with PSO, and PSA sine PSO.

**Material and Methods:**

Patients with PSO, PSA with PSO, and PSA sine PSO were evaluated and compared in a cross-sectional single centre study. Nail and enthesis abnormalities were evaluated by HFUS using high frequency probes (27 MHz). After a descriptive assessment, Brown University Nail Enthesis Scale (BUNES) and Madrid Sonography Enthesitis Index (MASEI) were used to assess nail and enthesis, respectively.

**Results:**

Fifty-nine patients were enrolled (19 PSO, 22 PSA with PSO, 18 PSA sine PSO). In patients with PSO and in those with PSA and PSO, HFUS evaluation identified the following nail alterations characterised by thickened matrix, inhomogeneous echogenicity of the nail bed, and increased blood flow by power Doppler. In 38.9% patients with PSA sine PSO, a subclinical nail involvement was described. No difference was observed comparing BUNES values in three groups. In PSA patients with PSO and in those with PSA sine PSO, HFUS assessment of entheses mainly showed a hypoechoic aspect and thickness of the tendon, focal cortical erosion, and ossification. A subclinical enthesis involvement in 47.4% patients with PSO was observed. No difference was reported comparing MASEI values in three groups.

**Conclusion:**

Qualitative and quantitative abnormalities of nail and enthesis were demonstrated by HFUS in patients with PSO, PSA with PSO, and PSA sine PSO, suggesting a practical additional tool to be used in clinical settings. Furthermore, HFUS highlighted a subclinical nail involvement in patients with PSA sine PSO and enthesis subclinical alterations in patients with PSO.

## Introduction

In last years, the concept of psoriatic disease has been developed thanks to a better understanding of molecular pathogenetic mechanisms leading to psoriasis (PSO) and associated musculoskeletal manifestations [1]. PSO is a common chronic skin inflammatory condition associated with quality-of-life impairment and disability [2, 3]. The disease presents cutaneous manifestation as erythematous and scaling plaques with prototypical distribution in plaque-type PSO, but also rarer and severe variants, such as pustular and erythrodermic PSO and special localization such as nails, genitals, skin folds, and palmo-plantar areas [4, 5]. Independently from the cutaneous phenotypical variability, a percentage of patients with PSO may develop psoriatic arthritis (PSA), variously characterized by the involvement of peripheral joints, entheses, synovial sheaths of tendons, and axial skeleton [6]. PSA may also be observed in patients without PSO but with a family history positive for PSO in first or second-degree relatives, defined as PSA sine PSO [6]. In this context, the involvement of nails is one of the hallmarks and diagnostic clues of PSO, esteemed to be present in up to 70% of patients affected by PSO [7]. It may involve all the nail components and may predict the evolution from cutaneous to musculoskeletal involvement [8]. Furthermore, the involvement of synovio-entheseal complex and the enthesis-related inflammation may be considered as an early event in PSA and its diagnostic usefulness has been pointed out [9]. Thus, as described in other rheumatic diseases [10–12], the role of imaging features is increasingly suggested in identifying those clinically silent abnormalities which may predict the development of PSA in PSO patients and may help to early diagnosis of the disease for a timely treatment [13–15]. On these bases, musculoskeletal ultrasonography with the use of high frequency probes (HFUS) and power Doppler (PD) may be suggested in determining subclinical inflammation of the area under ultrasound examination [16–18].

In this study, we aimed at performing a qualitative and quantitative characterization of nail and enthesis abnormalities using HFUS in patients with PSO, PSA with PSO, and PSA sine PSO.

## Patients and methods

### Clinical features

In this cross-sectional single centre study, patients were selected among those consecutively admitted to the Dermatologic and/or Rheumatologic Clinics of University of L’Aquila at San Salvatore Hospital, L’Aquila, Italy, from June to December 2021. All patients were assessed if fulfilled widely available criteria for PSO or PSA classification criteria [19, 20].

Three groups of patients were assessed: (i) patients affected by PSO but without any musculoskeletal involvement (PSO group); (ii) patients affected by PSA and evident skin involvement by PSO (PSA with PSO group); (iii) patients affected by PSA but without any skin involvement by PSO (PSA sine PSO group). Voluntary adult patients (> 18 years) with a minimum disease duration of 12 months were considered in this study. Patients were excluded if treated with ongoing systemic immunomodulating therapies for PSO and/or PSA, insufficient wash-out period from previous systemic immunomodulating therapies, and/or other medical conditions potentially influencing nail and enthesis conditions. An adequate wash-out period from systemic immunomodulating therapies was codified as the discontinuation for a time of at least 3 half-lives of the administered drug.

Experienced dermatologists and rheumatologists performed the clinical evaluation. The Psoriasis Area and Severity Index (PASI) [21] was used to measure severity and extension of PSO and the specific Nail Area and Severity Index (NAPSI) for nail involvement [22]. Tender and swollen joint counts, and Leed Enthesitis Index (LEI) were used to evaluate the features of PSA [23].

Ethical approval was obtained from the Internal Review Board of the University of L’Aquila (protocol number Internal Review Board University of L’Aquila 01/2022). Moreover, patients signed an informed consent allowing the use of clinical records for scientific purposes.

### HFUS features

HFUS was carried out at the Radiology Department of the University of L’Aquila using high frequency probes (27 MHz), available on US machines (Esaote my Lab X8), having available a software implementation improving the quality of ultrasound imaging with Color Doppler. The imaging parameters for Doppler ultrasound examinations were set to increase the detection of low-velocity, low-volume flows within the inside of the nail bed and tendon enthesis. The PD specifications have been standardized through a frequency ranging from 10.0 to 12.5 MHz and pulse repetition frequencies ranging from 0.9 to 1.0 kHz; color gain was set avoiding excessive color noise (color vs. echo priority ranging from 40 to 60% and color persistence adjusted to high values).

Ultrasound assessment of nail abnormalities was performed evaluating hands in all subgroups of patients. All ten nails of the hands were imaged. Longitudinal scan identified nail plate, composed by two hyperechoic lines representing the dorsal and ventral areas with a virtual anechoic space between them; nail bed a hypoechoic band between the superior hyperechoic nail plate and the inferior hyperechoic distal phalanx; nail matrix visualized as an isoechoic region under the proximal nailfold at the proximal portion of the nail bed. Brown University Nail Enthesis Scale (BUNES) was used to evaluate different nail structures and PD activity [24]. Morphologic normal findings were indicated with a score of 0 for each area scanned (nail plate, matrix, and bed). Nail plate changes (focal hyperechoic areas of the ventral plate/irregular border of ventral and dorsal plate), abnormal or thickened nail beds (2.0–3.0 mm) and/or matrix were scored as 1. Nail bed and matrix PD findings were assessed by 0 = no signal, 1 = confluent signal in < 25% of the area, 2 = confluent signal in > 25% and < 50%, 3 = confluent signal > 50%. A mean score of BUNES morphometry of 1.5 and a mean score of BUNES PD of 3 were used as threshold to identify a pathologic nail involvement. In addition, an ultrasound evaluation of enthesis abnormalities was provided in all subgroups of patients. Madrid Sonography Enthesitis Index (MASEI) score was used to assess entheses, exploring six sites bilaterally: proximal plantar fascia, distal Achilles tendon, distal and proximal patellar tendon insertion, distal quadriceps tendon and distal brachial triceps tendon [25]. The evaluation of the tendon thickness was based on the subjective measurement of the assessor. Each tendon was scanned in both the longitudinal and transverse planes. The HFUS evaluation was performed for each enthesis about the following features: calcifications, bursae, erosions, thickness, and structure of tendon (cortical bone profile, intratendon and paratendon echogenicity) and PD signal in bursa or enthesis. Each item was scored as 1 point, except for calcification (0 = absent, 1 = small calcification or ossification with an irregularity of enthesis cortical bone profile; 2 = clear presence of enthesophytes; 3 = large calcifications or ossifications) erosion, and PD signal (0 or 3 whether they are present or not) [25]. As reported in available literature [26], a cut-off of 4.5 of MASEI was used to codify a pathologic enthesis involvement.

### Statistical analysis

Mean BUNES and MASEI scores derived by HFUS assessment of nail and enthesis abnormalities were compared among patients with PSO, PSA with PSO, and PSA sine PSO by using the one-way analysis of variance (ANOVA) test. Two-sided P values < 0.05 were considered as being statistically significant.

## Results

### Clinical features of evaluated patients

In this study, 59 patients (17 males/42 females) were included, belonging to the three groups, 19 affected by PSO, 22 by PSA with PSO, and 18 by PSA sine PSO. On clinical dermatologic evaluation, PASI and NAPSI characterized patients affected by PSO and those by PSA with PSO. The most common clinical PSO variant was plaque-type PSO which was present in 94.7% and 81.6% in patients affected by PSO and in those by PSA with PSO, respectively. Nail PSO was present in 68.4% and 81.8% in patients with PSO and in those affected by PSA with PSO, respectively. The clinical rheumatologic evaluation showed that patients with PSA with PSO and PSA sine PSO were characterised by an increased number of tender and swollen joints, as well as by LEI. Clinical findings of evaluated patients are reported in Table 1.

**Table 1:** Descriptive features of assessed patients

	PSO	PSA with PSO	PSA sine PSO
Patients	19	22	18
*Demographic features*			
Age, years	50.2 ± 14.3	56.5 ± 11.4	56.2 ± 1.5
Male Sex	47.4%	22.7%	16.7%
BMI	26.9 ± 6.8	27.7 ± 5.5	25.6 ± 3.2
*Dermatologic assessment*			
PASI	7.9 ± 6.2	3.4 ± 4.8	0.0
Plaque type PSO	94.7%	81.6%	0.0%
Inverse/genital PSO	31.6%	4.5%	0.0%
Palmo-plantar PSO	5.3%	18.2%	0.0%
Guttate PSO	31.6%	9.1%	0.0%
Nail PSO	68.4%	81.8%	0.0%
NAPSI	15.3 ± 12.3	23.2 ± 16.6	0.0
*Rheumatologic assessment*			
Tender joints	2.0 ± 3.9	9.0 ± 7.0	9.5 ± 7.1
Swollen Joints	0.0	0.4 ± 0.1	0.7 ± 0.5
LEI	0.0	1.5 ± 0.6	1.4 ± 0.8
*Ultrasound evaluation*			
BUNES nail morphometry	1.5 ± 0.4	1.6 ± 0.6	1.3 ± 0.5
BUNES nail PD	1.9 ± 1.9	2.5 ± 2.1	2.4 ± 2.1
MASEI	4.6 ± 3.7	6.5 ± 3.6	4.9 ± 2.9

Continuous variables are expressed ad mean and standard deviation, dichotomic variables are expressed as percentage

*PSO* psoriasis, *PSA* psoriatic arthritis, *BMI* body mass index, *PASI* Psoriasis Area and Severity Index, *NAPSI* Nail Area and Severity Index (NAPSI), *LEI* Leed Enthesitis Index, *MASEI* Madrid Sonography Enthesitis Index, *BUNES* Brown University Nail Enthesis Scale, *PD* power doppler

### HFUS nail evaluation

**Fig. 1:**
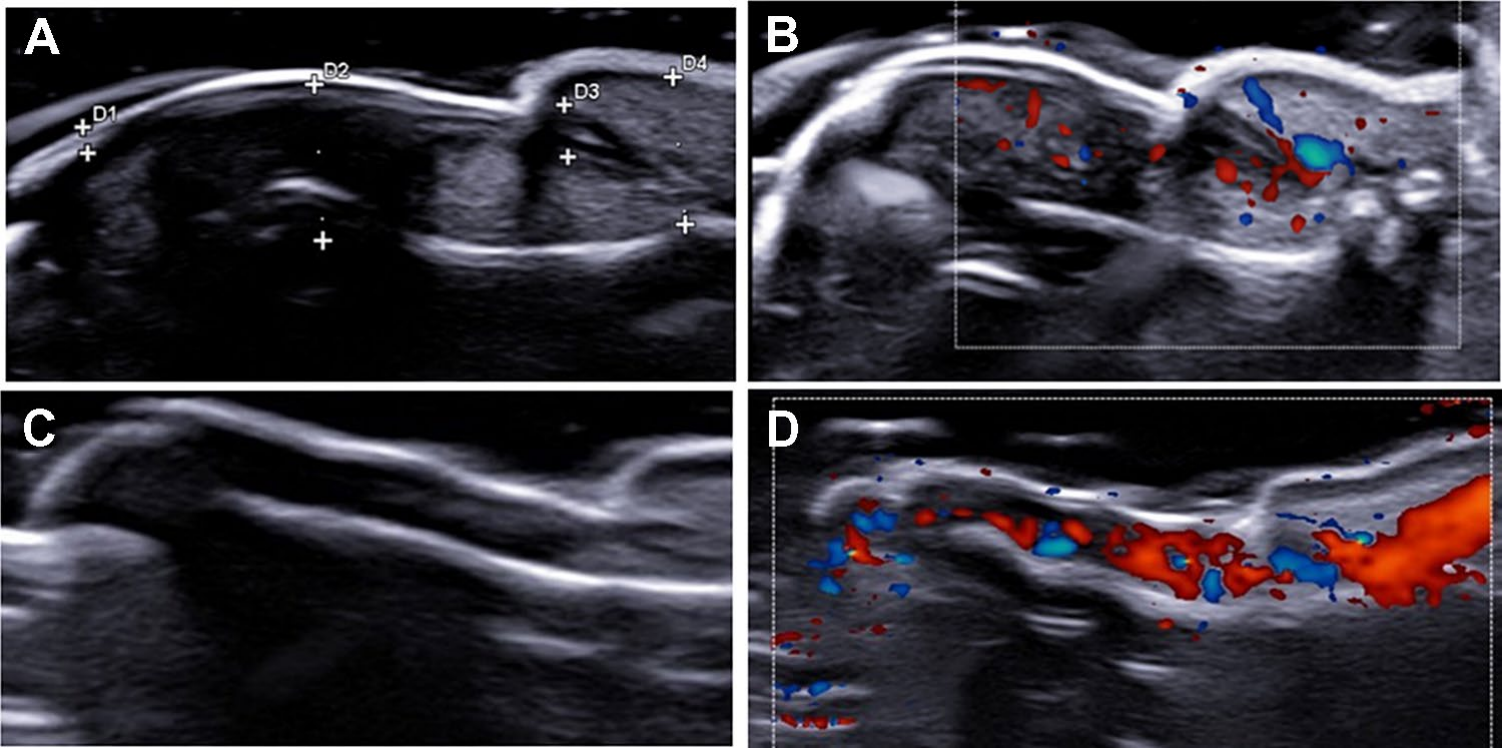
HFUS nail evaluation. PATIENT 1: **A** structural nail changes characterized by inhomogeneous echogenicity nail bed (d1), enlarged nail enthesis (d2), thickened matrix (d4) and plate (d3) with overall increased blood flow (**B**); PATIENT 2: **C** normal nail morphology without and with an increased Doppler signal (**D**)

Descriptively, HFUS evaluation identified different morphological and vascular nail findings in the three groups. In patients with PSO, the structure of nail was mainly preserved but an increased blood flow, identified by PD, was found as elongated, dilated, and tortuous blood vessels because of an active inflammatory process. Assessing the nails in patients with PSA with PSO, HFUS evaluation showed some structural changes. Thickened matrix, inhomogeneous echogenicity nail bed, enlarged nail enthesis, increased blood flow, loss of ventral and dorsal plate definition, were observed in this group of patients. These findings are shown in Fig. 1. The assessment of patients with PSA sine PSO showed a less marked evidence of vascular and echogenicity changes in the evaluated nails by HFUS. However, 38.9% of patients with PSO sine PSA were codified as having a subclinical involvement of nails, characterised by the presence of nail hyperechoic areas and thickened beds associated with PD findings in nail bed and matrix. The nail ultrasound abnormalities were also scored by BUNES. No significant difference was reported comparing the three groups of patients based on the mean values of BUNES morphometry (*p *= 0.201) and BUNES PD (*p *= 0.391).

### HFUS enthesis evaluation

**Fig. 2:**
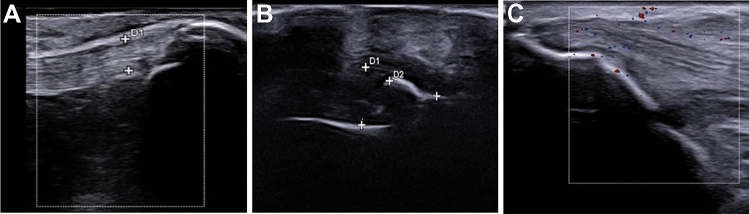
HFUS enthesis evaluation. **A** Thickened distal rotuleus tendon (d1: 4.5 mm) with irregular cortical profile without significant increase in the vascular pattern; **B** Thickened Achilles tendon (d1: 5.6 mm) with large calcification (d2; MASEI score = 3); **C** Normal fibrillar pattern of triceps tendon without significant Doppler signal

HFUS evaluation of entheses showed overlapping alterations in patients with PSA with PSO and in those with PSA sine PSO. In these groups, modifications of the structure of the entheses have been identified as a hypoechoic aspect and thickness of the tendon, focal cortical erosion, and enthesis ossification. No significant difference was noticed assessing the two sides of each scanned tendon. However, an increase of vascularity was rarely reported by PD assessment. These findings are shown in Fig. 2. In 52.6% of patients with PSO, a normal enthesis insertion with normal fibrillar pattern and bone profile was observed. Whereas in 47.4% of these patients with PSO without a clinically relevant musculoskeletal involvement, some alterations of entheses were reported by HFUS. Altered echogenicity of tendon structure, irregularity of enthesis cortical bone profile, and enthesophytes were observed despite normal values of LEI, which were retrieved in these patients. MASEI was also employed to score the enthesis abnormalities and no significant difference was reported comparing the three groups of patients (*p *= 0.166).

## Discussion

In our single centre cross-sectional study, the potentiality of HFUS was explored in evaluating nail and enthesis abnormalities in patients with PSO, PSA with PSO, and PSA sine PSO. HFUS may be considered as the next frontier in ultrasound evaluation. It may have many clinical applications increasing diagnostic potentiality and evaluating the efficacy of administered therapies. In addition, the relevance of this noninvasive imaging tool may highlight the possibility to reduce the need of invasive investigations. By using HFUS, we provided a qualitative and quantitative characterization of nail and enthesis abnormalities in patients with PSO, PSA with PSO, and PSA sine PSO.

In patients affected by PSO and in those by PSA with PSO, HFUS evaluation identified some nail alterations characterised by thickened matrix, inhomogeneous echogenicity of nail bed, and increased blood flow by PD assessment. In fact, ultrasonography may provide valuable information regarding the morphostructural changes and PD in nails in patients with PSO and PSA [27]. This technique may identify nail alterations in patients with PSO and PSA, with or without a relevant clinical involvement, when compared with healthy control [28–30]. Therefore, ultrasonography may allow the clinicians to assess the presence of nail changes offering the possibility to score the severity of the inflammatory lesions, thus suggesting its clinical usability as diagnostic and prognostic tool [31, 32]. In addition, in our patients with PSA sine PSO, a subclinical nail involvement was also observed overlapping with what reported in PSA with PSO and in those with PSO considering the values of BUNES. These data could suggest a nail subclinical involvement in some patients with PSA sine PSO, which could predict the occurrence of skin involvement. It has been reported that rheumatological manifestations may precede the clinical presence of cutaneous involvement in 20% of patients with PSA sine PSO [33]. Furthermore, this subclinical nail involvement could reinforce the concept of psoriatic disease continuum, ranging from skin involvement to the musculoskeletal manifestations [34]. According to the nail-entheseal pathogenesis proposed in psoriatic disease [1, 9, 35], the nail lesions are frequently associated with the presence of enthesis involvement suggesting the relevance of their evaluation in managing these patients [36–38].

In our cohort, HFUS assessment of entheses showed modifications of these structures mainly characterised by a hypoechoic aspect and thickness of the tendon, focal cortical erosion, and ossification. These features were mainly observed in patients affected by PSA with PSO and in those by PSA sine PSO. However, our preliminary data could also propose a subclinical enthesis involvement in patients with PSO overlapping with what observed in PSA with PSO and in PSA sine PSO as per results of MASEI. These results may provide further insights in this context by using HFUS suggesting that enthesis abnormalities may be highlighted in a substantial proportion of patients with PSO who are asymptomatic for musculoskeletal involvement [39–42]. Therefore, a more accurate assessment of a subclinical enthesis involvement, provided by HFUS, could possibly answer to the question if these imaging features could predict the risk of future clinically relevant musculoskeletal inflammation in these patients.

Taking together all these findings, HFUS may be added to an integrated and multidisciplinary approach of the psoriatic disease. The combination of clinical, both dermatological and rheumatological, assessment with HFUS may provide the ideal environment for a prompt assessment, immediate discussion, and an optimum management for each patient.

Although providing some insights of HFUS potentiality in patients with psoriatic disease, some limitations, mainly related to the single centre design and the relatively small sample size, should be considered in limiting the external validity of our data. Thus, subsequent confirmatory studies are needed to fully confirm these data and to evaluate their clinical relevance. The predictive role of these imaging abnormalities should be properly evaluated in specific designed longitudinal studies. Finally, an additional limitation of the present work could be that a single operator was involved in HFUS assessment, therefore the possible inter-observer variability of the ultrasonography findings should be entirely clarified in additional specific designed studies.

In conclusion, qualitative and quantitative abnormalities of nail and enthesis were demonstrated by HFUS in assessed patients with PSO, PSA with PSO, and PSA sine PSO, suggesting a practical and reliable tool to be used in clinical settings. Furthermore, HFUS highlighted a subclinical nail involvement in patients with PSA sine PSO and some enthesis subclinical alterations in patients with PSO. Although further studies are needed, our findings may offer the opportunity to better characterize the phases of the psoriatic disease by a multidisciplinary approach combining dermatological and rheumatological assessments with HFUS.

## Data Availability

All the generated data are included in the body of the article.
